# Supplementary material for: Blenderised Tube Feeds vs. Commercial Formula: Which Is Better for Gastrostomy-Fed Children?
Source: Nutrients. 2022 Jul 29;14(15):3139. doi: 10.3390/nu14153139 (PMC9370549; doi:10.3390/nu14153139)
Supplement: Supplementary file 1 [file nutrients-14-03139-s001.zip › nutrients-1799531-supplementary.pdf]

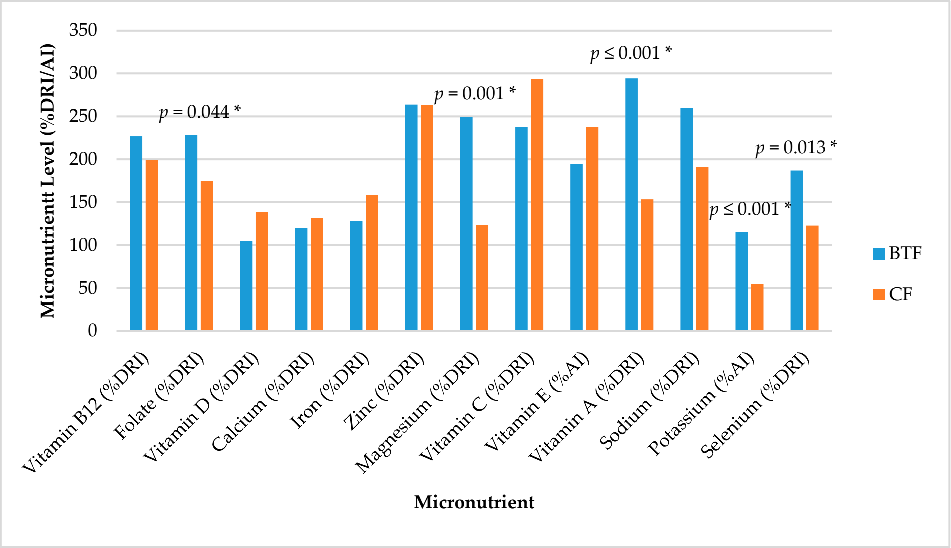

Figure S1. Comparison of Micronutrient content of blenderised tube feeds (BTF) and commercial formula (CF) Diets. AI, Adequate Intake; RDI, Recommended Dietary Intake. Results are mean %RDI or %AI. * Statistically significant ($p < 0.05$).

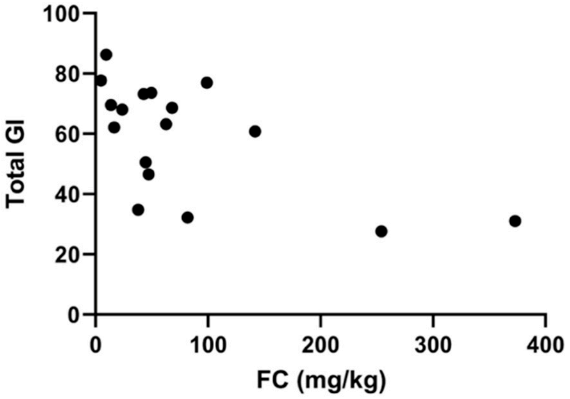

Figure S2. Correlation between fecal Calprotectin (FC) levels and total GI-Peds-QL questionnaire results. GI-Peds-QL, Paediatric Quality of Life Inventory Gastrointestinal Symptoms Scale; GI, gastrointestinal.
